# Perivascular fat imaging by computed tomography (CT): a virtual guide

**DOI:** 10.1111/bph.15634

**Published:** 2021-09-23

**Authors:** Christos P. Kotanidis, Charalambos Antoniades

**Affiliations:** ^1^ Division of Cardiovascular Medicine, Radcliffe Department of Medicine University of Oxford Oxford UK; ^2^ Acute Vascular Imaging Centre, Investigational Medicine University of Oxford Oxford UK

**Keywords:** atherosclerosis, attenuation, CT imaging, inflammation, perivascular adipose tissue, radiomics

## Abstract

Imaging in medicine has been revolutionised by technological, computational and research advances over the past decades. Computed tomography (CT), in particular, has seen rapid evolution especially in the field of cardiovascular non‐invasive imaging. It is being recognised as the first‐line tool for the assessment of stable and unstable disease with diagnostic, prognostic and re‐stratification potential. Vascular inflammation is a key component of the atherosclerotic process and has been shown to induce molecular, transcriptional and structural changes to perivascular adipose tissue (PVAT). Being a diverse structure itself, PVAT surrounds the human vessels and is characterised by a highly rich secretome, including, amongst others, adipokines, cytokines, gaseous messengers and miRNAs It is implicated in a bidirectional interplay with the adjacent vascular wall, affecting and being affected by aspects of its biology, mainly inflammation. In this review, we discuss the current status of cardiac CT in imaging vascular inflammation through PVAT phenotyping.

**LINKED ARTICLES:**

This article is part of a themed issue on Molecular imaging ‐ visual themed issue. To view the other articles in this section visit http://onlinelibrary.wiley.com/doi/10.1111/bph.v178.21/issuetoc

**Abbreviations**
CCTA: coronary CT angiography
eNOS: endothelial NOS
FAI: fat attenuation index
FRP: fat radiomic profile
PVAT: perivascular adipose tissue

## 1 INTRODUCTION

Medical imaging has experienced rapid advances in recent years alongside technology and analytics. In the field of cardiology, non‐invasive imaging is of paramount importance, with cardiac CT now recognised as the central diagnostics tool, for disease assessment and cardiovascular risk stratification. Thanks to its wide use and availability to clinical practice, there is the expected rapid growth in imaging datasets accessibility, which can be utilised for addressing the unmet need for new biomarkers in the battle to treat and prevent coronary artery disease (CAD).

Coronary artery disease remains a significant contributor to mortality globally despite the fact that advances have been made in primary and secondary prevention (Antoniades & Harrison, 2019; Kalbacher et al., 2018), the so‐called residual cardiovascular risk persists (Ferrari & Catapano, 2016; Liberale et al., 2020). Many clinical trials utilising novel, effective but at the same time costly, therapies such as anti‐proprotein convertase subtilisin/kexin type 9 (PCSK9) monoclonal antibodies (Schmidt et al., 2017) or antibodies against chemokines (Ridker et al., 2017) have been successful in targeting residual cardiovascular risk. However, in the field, we are still lacking early and accurate detection of the most vulnerable patients who would benefit the most from these new and expensive therapeutics.

In this visual review, we elaborate on the emergence of non‐invasive phenotyping of perivascular adipose tissue (PVAT) using CT as a promising marker for early detection of vascular inflammation and the atherogenic process. We summarise the basic biology of PVAT and its contribution to atherosclerosis, with special focus on its bidirectional interplay with the vascular wall. We describe the perivascular fat attenuation index (FAI_PVAT_) and discuss the diagnostic and prognostic value of macroscopic adipose tissue radiomic characterisation.

## 2 PERIVASCULAR ADIPOSE TISSUE (PVAT) DEFINITION

Human adipose tissue is distributed anatomically into two major compartments comprising of the subcutaneous fat (SAT) and visceral fat (VAT). Functionally, adipose tissue can be white (WAT), brown and ‘beige’ or ‘brite’(Kotanidis & Antoniades, 2020). White acts mainly as metabolic energy storage, brown adipose tissue is primarily tasked with non‐shivering thermogenesis, while the term ‘beige’ refers to brown‐like adipocytes inside white that has the ability to produce heat via uncoupling of mitochondrial respiration (Mancio et al., 2018).

**FIGURE 1:**
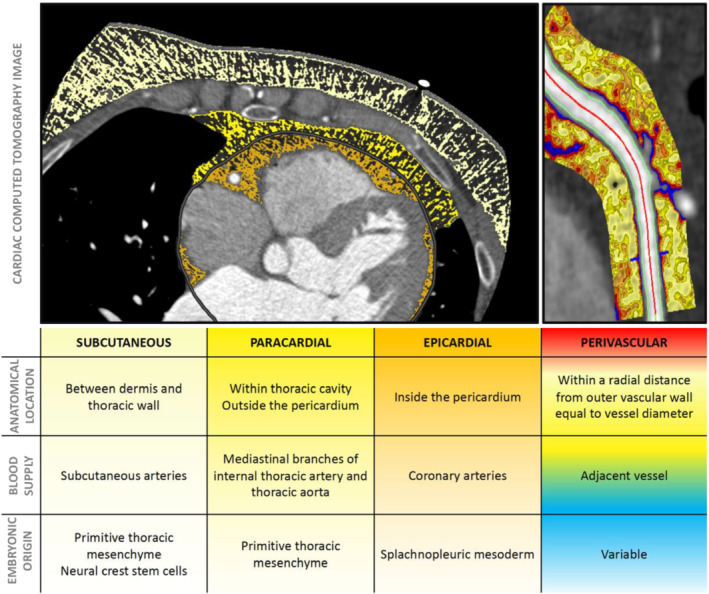
Presentation of the four different adipose tissue depots within the thorax from an axial cross‐sectional slice taken from a coronary CT angiogram image

Within a cardiac CT image, we come across four distinct types of thoracic adipose tissue: ‐ subcutaneous, paracardial, epicardial and perivascular (Figure 1). Epicardial adipose tissue (EAT) is white and found between the myocardium and the visceral layer of the pericardium. It differs embryologically from pericardial (or paracardial) fat, which is the part of intrathoracic visceral fat on the outside of the parietal layer of the pericardium (Iacobellis, 2015). Epicardial adipose tissue originates from the splachnopleuric mesoderm, along with the heart and is supplied with blood from the coronary circulation (Antonopoulos & Antoniades, 2017). Paracardial fat is part of the primitive thoracic mesenchyme and is vascularised by noncoronary arteries (Iacobellis, 2015). PVAT is defined as any adipose tissue located within a radial distance from the outer vessel wall equal to the diameter of the adjacent coronary vessel (Antonopoulos et al., 2017). This definition underlines the importance of biology, as opposed to crude anatomical borders, in defining distinct fat depots.

Functionally, PVAT is a highly dynamic and metabolically active tissue surrounding most vascular beds in the human body, with the exception of neural and pulmonary vessels (Aghamohammadzadeh et al., 2012). In large vessels, PVAT is contiguous with the adventitial layer, whereas in small vessels and microvessels, PVAT adipocytes are essentially an integral part of the vascular wall itself. It contains predominantly white adipose tissue, but it has been reported to exert thermogenesis after exposure to cold (Akoumianakis & Antoniades, 2017). Healthy PVAT is composed of adipocytes, neural cells, stem cells, microvasculature and inflammatory cells. Understandably, its exact phenotype arises from the differential balance of the aforementioned elements, dependent on its anatomical location and localised or systemic pathologies present (Szasz & Webb, 2012).

## 3 VASCULAR WALL AND PVAT: A CLOSER LOOK INTO THE BIOLOGY

### 3.1 Signals from the outside: fat affecting the vasculature

**FIGURE 2:**
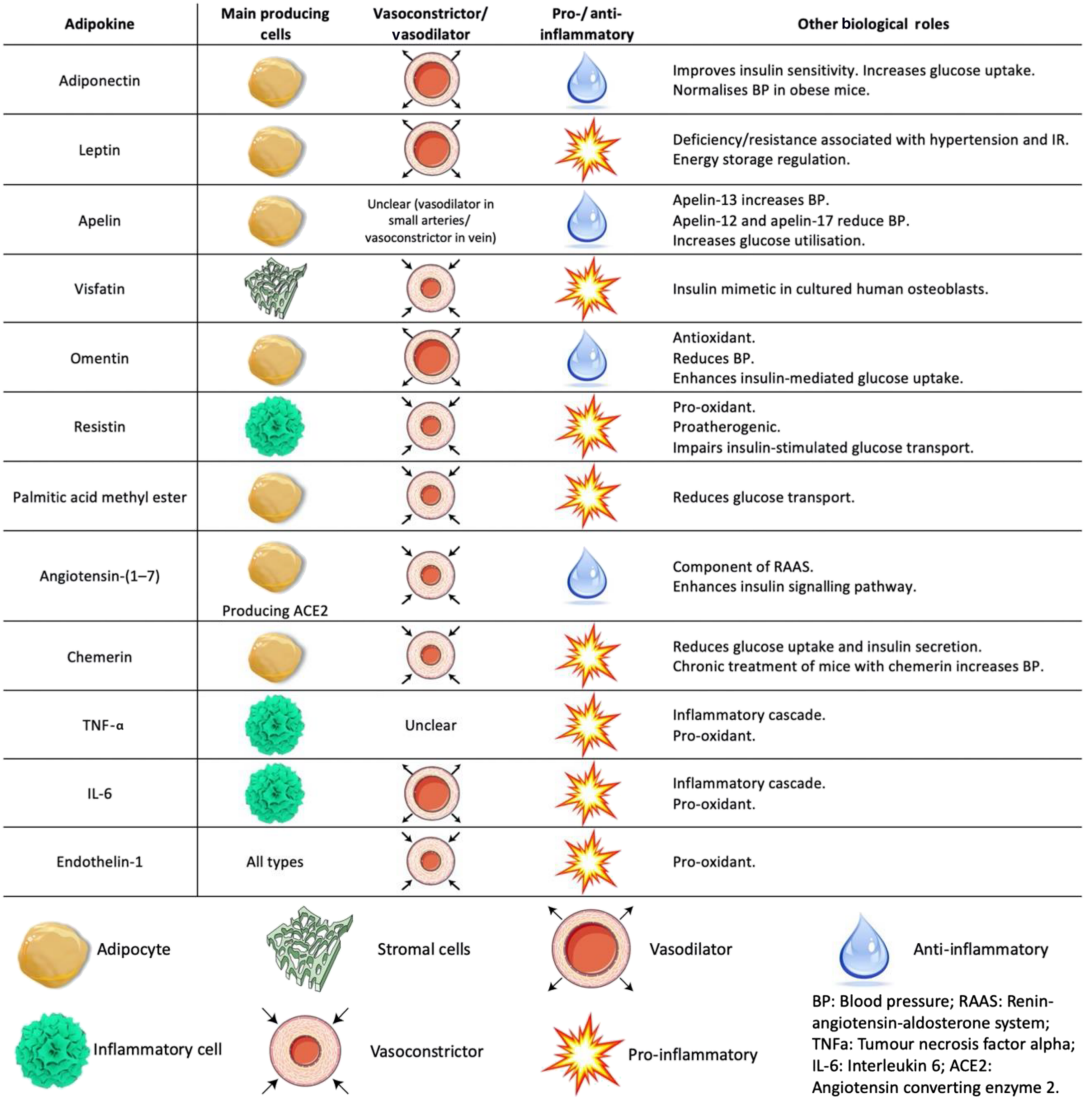
Main roles of key adipocytokines secreted by perivascular adipose tissue (PVAT). IR, insulin resistance; RAAS, renin–angiotensin–aldosterone system

Adipose tissue produces and secretes a range of bioactive molecules, including adipokines (such as adiponectin, leptin and apelin) and inflammatory cytokines, as well as microRNAs, microvesicles, inorganic molecules such as hydrogen sulfide, ROS and fatty acid metabolites. Figure 2 presents a summary of the main roles of key adipocytokines secreted by PVAT. These molecules can exert their function utilising either the endocrine or paracrine way. For instance, products from remote depots, such as the subcutaneous adipose tissue, can affect distant sites in the cardiovascular system in an endocrine manner, after being released into the bloodstream through the adipose tissue microvessels (Kotanidis & Antoniades, 2020). Given its close anatomical relation with vessels, PVAT may also exert direct effects on the adjacent vascular wall through the paracrine release of bioactive mediators. Furthermore, PVAT recruits a third form of signalling, namely, vasocrine signalling, in which PVAT‐derived adipokines are released through the underlying vascular wall and circulate in downstream microcirculation, potentially acting upon the entire vascular beds (Yudkin et al., 2005).

The effects of adipose tissue‐derived products on the vasculature are diverse, as they have been reported to influence vascular tone, inflammation, vascular smooth muscle cell migration, endothelial function and vascular redox state (Antoniades, Kotanidis, & Berman, 2019). First, PVAT facilitates the uptake and metabolism of vasoactive amines, such as noradrenaline (Ayala‐Lopez et al., 2017), acting as a natural anticontractile, whereas NOS produces and releases into the neighbouring vascular wall cells the gaseous messenger NO (Graham et al., 2018). Early studies on adiponectin have shown that it induces endothelial NOS (eNOS) phosphorylation through 5′‐AMP‐activated protein kinase (AMPK) or RAC‐alpha serine/threonine protein kinase (AKT)‐mediated mechanisms, increasing NO production and acting as a vasorelaxant (Margaritis et al., 2013). Vascular tone is also regulated by hydrogen sulfide (H_2_S) and palmitic acid methyl esters that activate ATP‐regulated and voltage‐gated potassium channels, respectively (King et al., 2014; Lee et al., 2011). Brown adipocytes have also been shown to produce aryl hydrocarbon receptor nuclear translocator‐like protein 1 (BMAL1), which controls angiotensinogen expression and therefore regulates angiotensin II levels and its effects on vasoactivity and BP (Chang et al., 2018). In addition, adiponectin and omentin can act as antioxidants by preventing the activation and membrane translocation of RAC1 and by down‐regulating the p22phox (also known as CYBA) regulatory protein, they inhibit the NOX1 and NOX2 isoforms of NADPH oxidases in the vascular wall, reducing superoxide production (Antonopoulos et al., 2015).

Conversely, leptin and resistin increase vascular oxidative stress, by activating NADPH oxidase isoforms (Margaritis et al., 2017). The coupling status of eNOS is also a determinant of superoxide production. When coupled it produces the beneficiary agent NO, but when uncoupled, it releases free oxygen species radicals. Chemerin, a multifaceted adipokine, reduces the bioavailability of tetrahydrobiopterin (BH_4_;sapropterin), which is an essential cofactor for eNOS, causing the enzyme to become mostly uncoupled and thereby decreasing NO production in favour of superoxide (Antoniades et al., 2006; Neves et al., 2014). PVAT may promote vascular inflammation by expression of endothelial cell adhesion molecules, which is induced by adipokines such as visfatin and adipose tissue‐derived pro‐inflammatory cytokines, such as IL‐1β and TNF (Oikonomou & Antoniades, 2018). Macrophage infiltration and polarisation within PVAT is an important determinant of its function in terms of cytokines and chemokines produced and released that are associated with vascular dysfunction (Lumeng et al., 2007). Furthermore, inflammatory cells may infiltrate the adventitia causing increased vasa vasorum neovascularisation, differentiation of fibroblasts to migratory myofibroblasts in a TGF‐β‐dependent manner and deposit of collagen (Maiellaro & Taylor, 2007).

**FIGURE 3:**
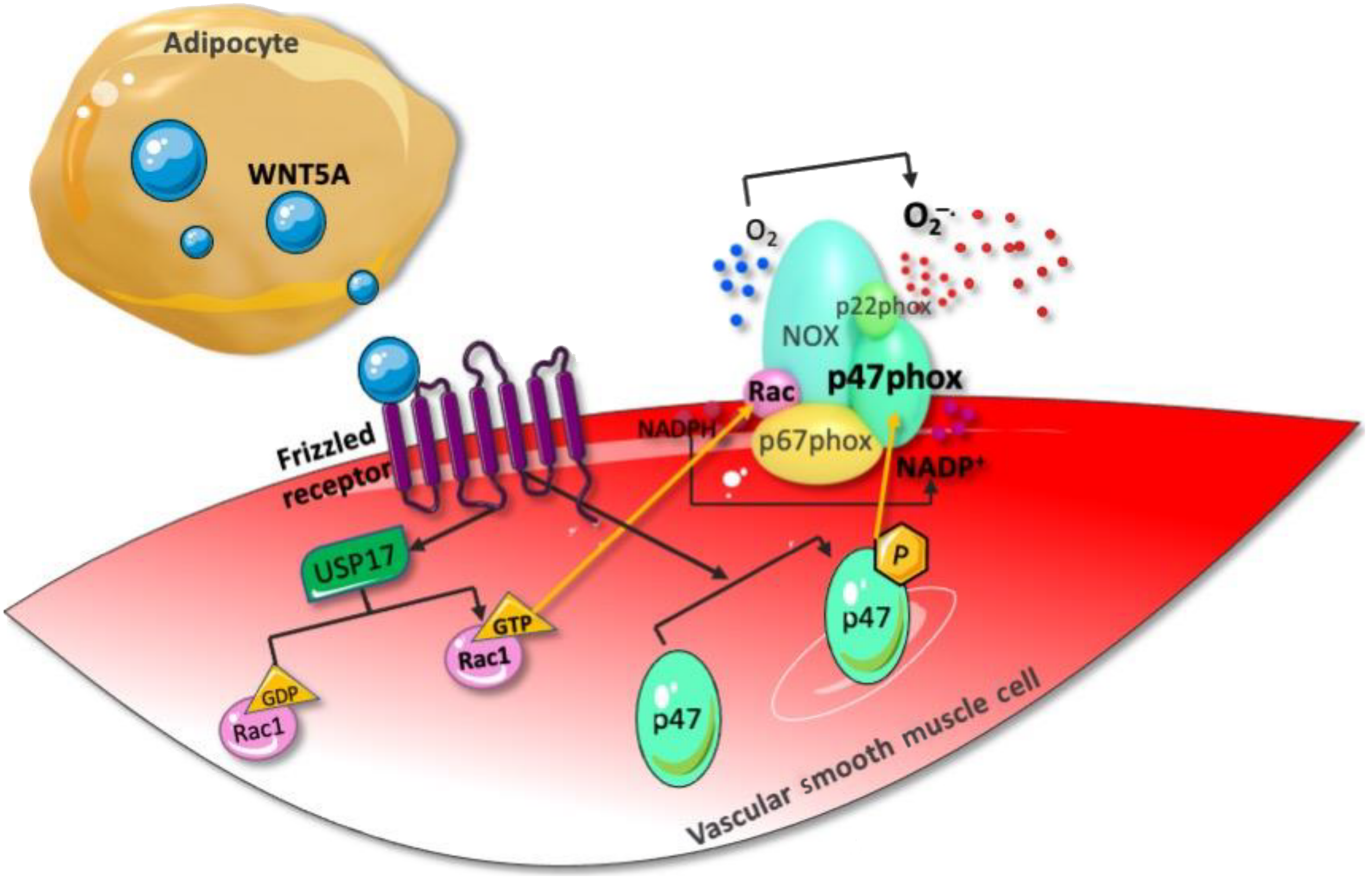
WNT5A is released from perivascular adipose tissue (PVAT) and binds to frizzled receptors on the arterial wall, activating the noncanonical wingless‐related integration site (WNT) signalling pathway. This leads to a USP17/RAC1‐mediated increase in NADPH oxidase activity and superoxide generation and induces endothelial dysfunction via endothelial NOS (eNOS) uncoupling

We have recently shown that a member of the wingless‐related integration site (Wnt) signalling pathway molecules, WNT5A (Wnt‐5a), is actively produced and secreted by PVAT, amongst other fat depots, and is involved in vascular redox balance in obese individuals (Akoumianakis et al., 2019). Circulating WNT5A levels in these patients are increased, with PVAT being one of the blood pools contributing tissues, whereas their arterial wall up‐regulates the expression of the WNT5A receptors FZD_5_. The noncanonical WNT signalling pathway is triggered within vascular smooth muscle cells, promoting the assembly of the NADPH oxidase complex in the membrane, increasing superoxide production and inducing endothelial dysfunction and eNOS uncoupling (Figure 3).

Apart from the hormones and peptides mentioned above, PVAT also secretes microRNAs, small RNA molecules involved in post‐transcriptional regulation. MicroRNAs are part of several processes, ranging from the recruitment of inflammatory cells to adipose tissue browning, myocardial fibrosis, atherosclerosis and vascular smooth muscle cell activation and their profile changes in response to obesity, insulin resistance and coronary heart disease (Fischer et al., 2017). Furthermore, exosomes released from PVAT are believed to polarise macrophages towards the M1 pro‐inflammatory profile, causing atherosclerosis in *ApoE* ^−/−^ mice (Xie et al., 2018). To summarise, PVAT acts as a dynamic regulator of vascular biology with outside‐to‐inside signalling that exerts both beneficial and detrimental contractile, inflammatory and oxidant effects to the adjacent vasculature.

### 3.2 Inside‐to‐outside signals: Vasculature affecting fat

#### 3.2.1 Systemic signals

The concept that adipose tissue is a dynamic tissue that interacts with other systems in the human body is well known, particularly when it comes to systemic signals (Table 1).

**TABLE 1:** Summary of the effects of inside‐to‐outside signals on adipose tissue

Systemic signals		
Signal	Effect on adipose tissue	Study
Cardiometabolic disease	• Hyperplasia • Hypertrophy	(Fuster et al., 2016)
	• Adipocytes acquire a dysfunctional, lipid‐laden phenotype	(Ketelhuth et al., 2019)
	• Inflammatory cell infiltration • Macrophage polarisation towards the M1 pro‐inflammatory phenotype	(Rutkowski et al., 2015)
	• Pericyte activation • Secretion of PDGF‐β • Localised neoangiogenesis	(Onogi et al., 2017)
	• Vasomotor dysfunction • Capillary rarefaction	(Chadderdon et al., 2014)
	• Hypoxia • Increased expression of hypoxia‐inducible factor‐1α • Fibrosis	(Pasarica et al., 2009)
	• Brown adipose tissue depletion	(Franssens et al., 2017)

Regional signals			
Signal	Model	Effect on perivascular adipose tissue	Study
Endovascular injury	Rodent	• Up‐regulation of MCP‐1, TNF‐α and IL‐6 and down‐regulation of adiponectin in periadventitial fat 1 day after the injury • Accumulation of F4/80‐positive macrophages and CD3‐positive T‐cells in periadventitial fat	(Takaoka et al., 2010)
Oxidative stress and release of peroxidation products	Human	• Up‐regulation of PPAR‐γ and adiponectin in perivascular adipose tissue around saphenous veins and internal mammary arteries	(Margaritis et al., 2013)
Angiotensin II‐induced up‐regulation of IL‐6, TNF‐α and IFN‐γ	Human	• Inhibition of preadipocyte differentiation • Proliferation of preadipocytes • Lower intracellular accumulation of lipid droplets • Attenuation shifts in CT imaging	(Antonopoulos et al., 2017)
Drug‐eluting stent‐induced hyperconstriction	Porcine	• Reduced adipocyte diameter • Increased CD68‐positive and IL‐1β‐positive cells • Increased ^18^F‐FDG uptake in *in vivo* PET‐CT imaging	(Ohyama et al., 2017)

Cardiometabolic disease, including obesity, insulin resistance and diabetes mellitus, causes shifts in adipose tissue phenotype (Fuster et al., 2016). In obesity, adipose tissue expands in terms of adipocyte number (hyperplasia) and/or size (hypertrophy) (Krotkiewski et al., 1983), with preadipocytes recruited in order to get differentiated into mature adipocytes. Hypertrophy on the other hand forces adipocytes to acquire a more dysfunctional, lipid‐laden phenotype (Ketelhuth et al., 2019). Fat gets infiltrated by an increasing number of inflammatory cells (Rutkowski et al., 2015) that drive macrophage polarisation from an M2 anti‐inflammatory phenotype, towards the M1 pro‐inflammatory type (Fujisaka et al., 2009), which is also involved in localised neoangiogenesis through secretion of platelet derived growth factor receptor beta (PDGFRβ) and activation of pericytes (Onogi et al., 2017). Increased expression of adhesion molecules (P‐selectin and E‐selectin) promotes further inflammatory cell infiltration, whereas vasomotor dysfunction and capillary rarefaction are also observed. In combination with the hypoxic micro‐environment, fibrosis is triggered, through increased expression of the hypoxia‐inducible factor 1α, that eventually promotes further its dysfunction and insulin resistance (Chadderdon et al., 2014; Pasarica et al., 2009). In addition, cardiometabolic disease is associated with reduced brown adipose tissue (BAT) volumes, abolishing its thermogenic and beneficial secretory properties (Villarroya et al., 2017). Finally, in response to signals from the periphery adipose tissue alters its secretome, with higher circulating levels of visfatin, chemerin, vaspin and resistin and lower levels of omentin and adiponectin amongst the observed changes in the setting of cardiometabolic disease (Antoniades, Kotanidis, & Berman, 2019).

It becomes clear that adipose tissue has the capability to sense and respond to signals originating from other tissue's pathologies. Chronic diseases, such as cancer, congestive heart failure, infectious and inflammatory disease (such as tuberculosis, rheumatoid arthritis, inflammatory bowel disease, etc.), as well as advanced age, result in cachexia (Tisdale, 2002). Cachexia pertains to a state of systemic chronic low‐grade inflammation, characterised by hyper‐catabolism leading to unintentional weight loss through lipolysis of adipose tissue (Han et al., 2018). Systemic low‐grade inflammation suppresses adiponectin production by fat and abolishes its insulin‐sensitising and antiatherogenic properties in humans with or without significant cardiovascular disease (CVD) (Antonopoulos et al., 2014). Therefore, it is evident that adipose tissue undergoes changes, ranging from expansion in the presence of high calorie intake seen in obesity, to atrophy found in conditions involving cachexia, to transcriptional alterations driven by circulating inflammatory molecules. This concept is being underlined by the so‐called obesity paradox. In a large‐scale meta‐analysis including over 30 million individuals, a U‐shaped or J‐shaped association was found between body mass index (BMI) and all‐cause mortality in the general population (Flegal et al., 2013). This observation, commonly known as the obesity paradox, can be interpreted if we take into account that, despite the fact that excess adipose tissue is associated with reduced survival, significantly reduced adipose tissue in chronic disease (such as cancer, heart failure, renal failure, chronic inflammatory diseases and other conditions) will also lead to poor survival rates. Therefore, it would be misleading to assume that only a positive association between BMI and short‐term (5–10 years) survival in the general population exists, as we would be ignoring all the negative effects of shrunken, dysfunctional adipose tissue in the presence of chronic disease (Antonopoulos & Tousoulis, 2017; Aune et al., 2016).

#### 3.2.2 Regional signals

**FIGURE 4:**
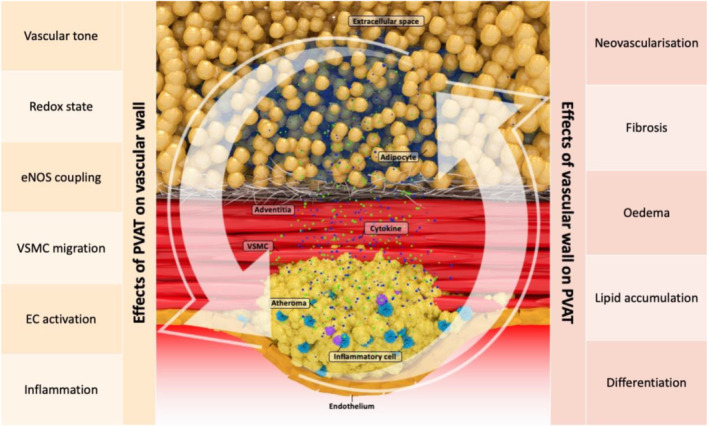
Illustration of the major components of the bidirectional interplay between the vascular wall and fat in the perivascular space. EC, endothelial cell; eNOS, endothelial NOS; PVAT, perivascular adipose tissue; VSMC, vascular smooth muscle cell

**FIGURE 5:**
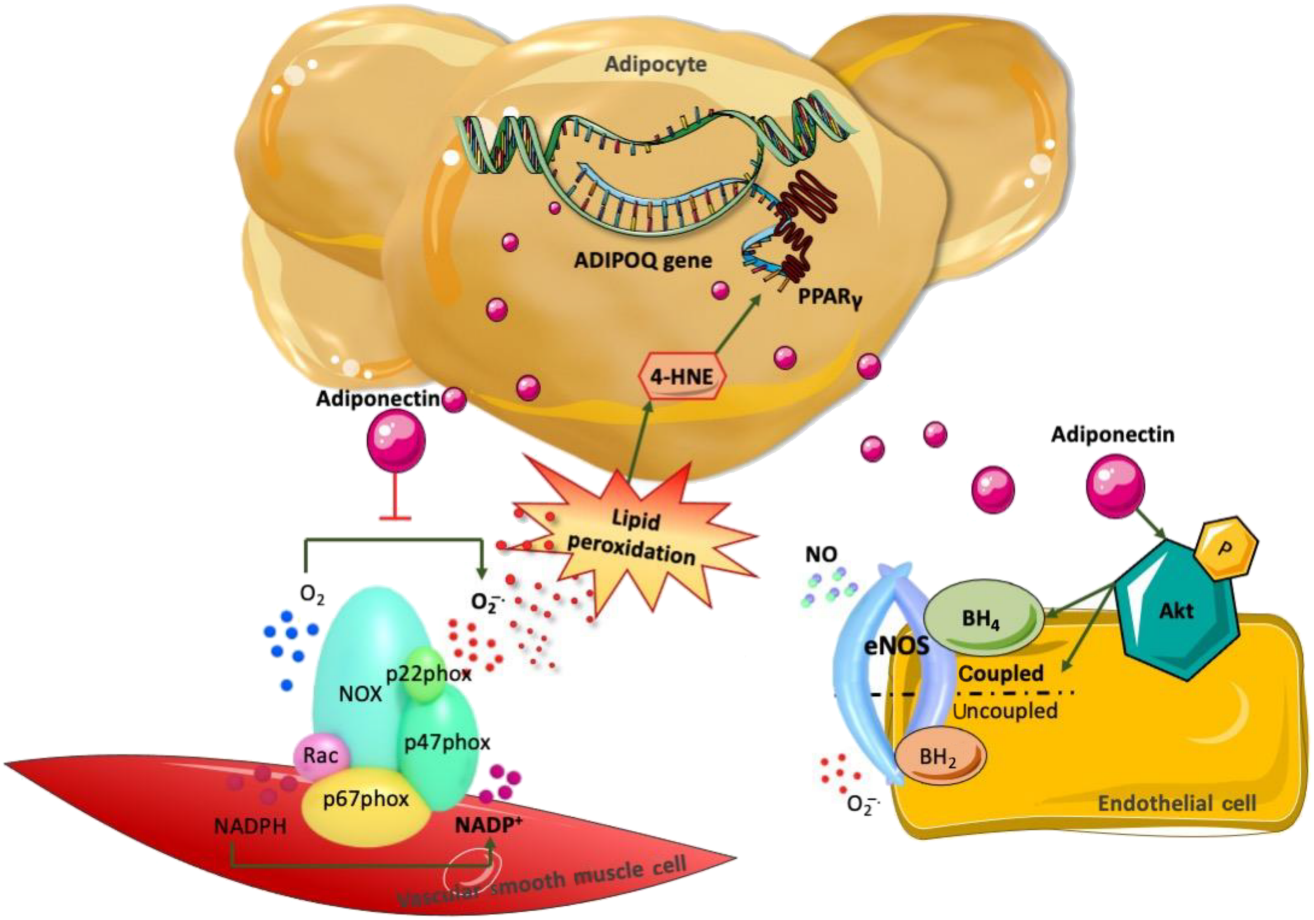
Enhanced production of superoxide leads to increased local production of lipid peroxidation products such as 4‐hydroxynonenal (4‐HNE), which travel into perivascular adipose tissue (PVAT) and up‐regulate adiponectin's gene expression via a PPARγ‐mediated mechanism. Adiponectin, in turn, is secreted and acts on vascular smooth muscle cells and endothelial cells by inhibiting the activity of NADPH oxidases as well as through Akt‐mediated stimulation of endothelial NOS (eNOS) activity and coupling, resulting in increased production of NO and reduced oxidative stress. ADIPOQ, adiponectin; BH_2_, dihydrobiopterin; BH_4_, tetrahydrobiopterin; O_2_^−^, superoxide

Apart from systemic signals, inside‐to‐outside signalling can occur in a localised manner via a bidirectional crosstalk loop between PVAT and the underlying vascular wall (Table 1). This concept has been introduced lately with important implications in our understanding of cardiovascular disease pathogenesis and diagnosis (Figure 4). Early studies underlined that overexpression of the p22phox subunit of the NOX1, NOX2 and NOX4 isoforms of NADPH oxidase in vascular smooth muscle cells exacerbates obesity and insulin resistance in mice fed on a high‐fat diet (Youn et al., 2014). In 2010, it was reported that up‐regulation of pro‐inflammatory adipocytokines, primarily TNF‐α, caused by intravascular injury (balloon‐induced or wire‐induced) in mice resulted in rapid phenotypic modifications of PVAT (Takaoka et al., 2010). A few years later, the term ‘adipose tissue sensing’ was introduced following observations that increased vascular oxidative stress, through free oxygen radical species, causes lipid peroxidation and creates by‐products, such as 4‐hydroxynonenal (4‐HNE), that diffuse into the perivascular space. In turn, adipocytes within the PVAT up‐regulate adiponectin's ADIPOQ gene expression in response to the presence of 4‐HNE via a PPAR‐γ (NR1C3)‐dependent mechanism. To conclude this protective ‘adipose tissue sensing’ loop, adiponectin is released back to the vessel wall, exerting its beneficiary antioxidant and vasoprotective effects (Margaritis et al., 2013) (Figure 5).

In addition, more recent studies have reported that inflammation in the human vascular wall triggers the release of inflammatory cytokines, such as TNF‐α, IL‐6 and IFN‐γ, which also diffuse into the perivascular space. PVAT responds by adopting a ‘cachexia‐type’ sequence of biological events within adipocytes, differentiation becomes suppressed and intracellular lipid formation decreases. Adipocytes get smaller in size as their intracellular lipid content depletes due to an increase in lipolysis and simultaneous decrease in adipogenesis. Both resulting from the adverse micro‐environment inside the perivascular space caused by vascular inflammation (Antonopoulos et al., 2017). Further to lipolysis and reduced adipogenesis, perivascular oedema may also appear around the inflamed artery as a result of increased inflammation‐induced permeability in the microcirculation and expansion of the interstitial space in the perivascular space. Finally, vascular inflammation has the potential to induce more permanent changes in the surrounding fat, such as fibrosis and neoangiogenesis, which are known to occur in the setting of chronic inflammation (Crewe et al., 2017). Indeed, inflammatory changes of coronary PVAT, as assessed *in vivo* by ^18^F‐fluorodeoxyglucose PET (^18^F‐FDG PET), were significantly enhanced at sites of coronary injury caused by everolimus‐eluting stents as compared with untouched control sites in an *in vivo* porcine model (Ohyama et al., 2017).

**FIGURE 6:**
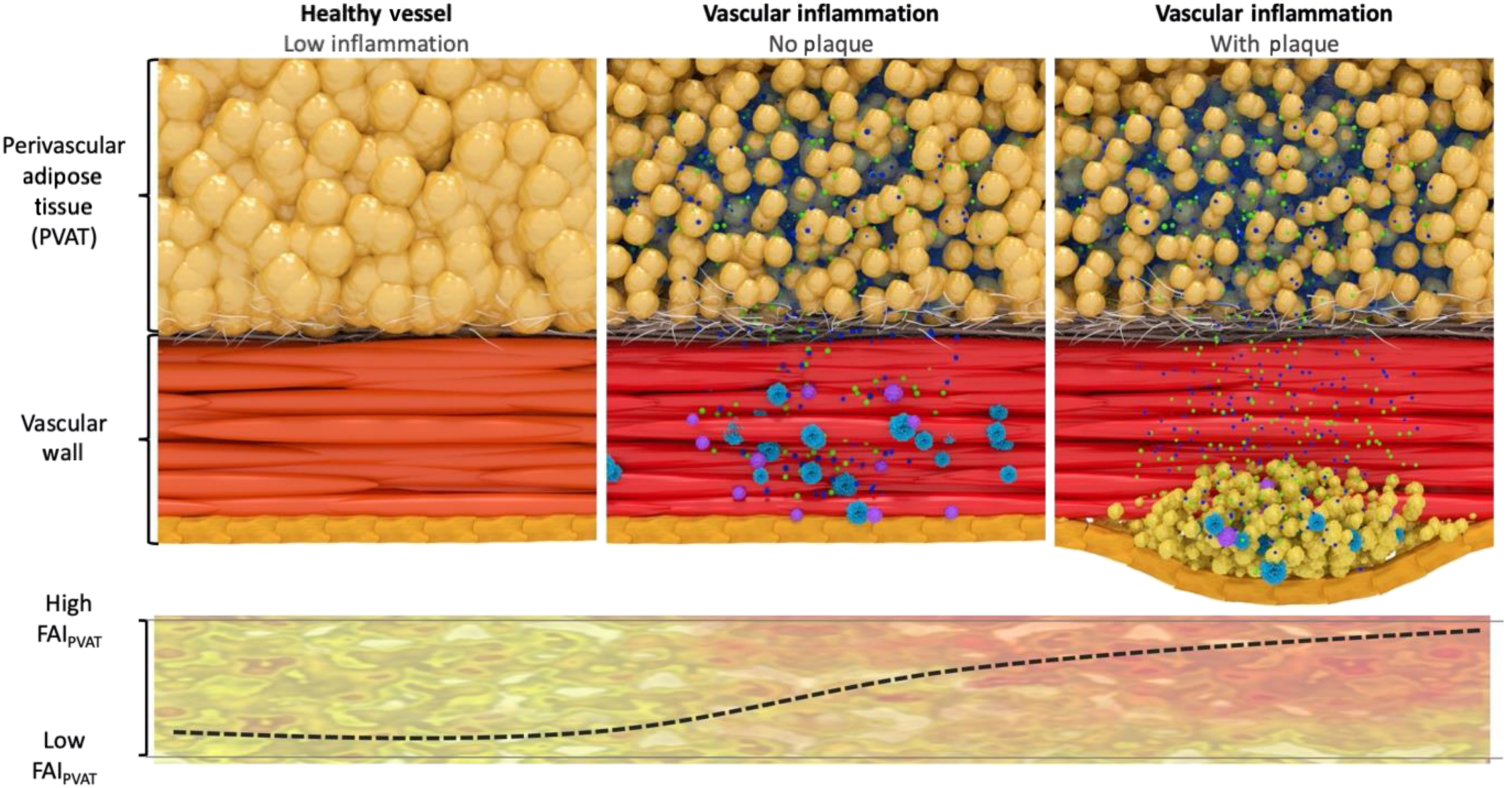
Morphological appearance of perivascular adipose tissue surrounding healthy and inflamed coronary arteries, depicting the gradient of adipocyte size induced by exogenous vascular inflammation

In summary, the above‐mentioned biological changes lead to the development of a gradient of adipocyte size around the inflamed human arteries. Smaller adipocytes can be found closer to the vascular wall, although as we move further away from the vessel, they gradually increase in size to eventually match the baseline size of the healthy fat adipocytes. At the same time, the interstitial space close to the wall of the vessel expands and becomes oedematous. Macroscopically, these changes are reflected by a smooth shift of the balance between the lipid and aqueous phase in the adipose tissue directly adjacent to the inflamed artery (Antonopoulos et al., 2017). As adipocytes get smaller in size through suppression of differentiation and decrease in intracellular lipid content, the lipid phase of the tissue starts to become depleted, and gradually, the aqueous phase reflecting localised oedema and increased interstitial space becomes more apparent (Figure 6). It should be noted though that, although these changes are dynamic and subject to the degree of coronary inflammation, their kinetics are not entirely understood.

## 4 PVAT ATTENUATION MAPS AND THE FAT ATTENUATION INDEX (FAI_PVAT_)

As evidenced from above, vascular inflammation affects PVAT, forcing it to adjust its biology and enter a state of hypercatabolism and suppressed adipogenesis, leading ultimately to macroscopic phenotypic alterations. Non‐invasive tracking of PVAT macroscopic changes has had a substantial impact in highlighting this adipose tissue depot as a very promising and applicable marker of disease presence in the clinical setting.

Conventional cardiac CT angiography is able to provide information on adipose tissue quality, by analysing attenuation shifts. CT has seen major developments during the past few decades, allowing for in‐depth three‐dimensional phenotyping of tissues, with excellent computer processing algorithms for segmentation and tissue characterisation. Cardiac CT in particular has experienced a renewed interest in the visualisation of cardiac disease, especially in the field of vascular inflammation and plaque characterisation (Antoniades, Kotanidis, & Berman, 2019). It can be combined with CT angiography for PVAT phenotyping and also with PET imaging for quantification of radiotracer uptake and provision of functional information on inflammatory and metabolic tissue activity. Recently, it has received a Class I indication by the European Society of Cardiology (ESC) guidelines as a first‐line investigation of suspected coronary artery disease (Knuuti et al., 2019), rendering it an imaging technique that is widely used and commonly available and will eventually dominate clinical practice in the field of cardiology. Table 2 presents a comparison of the most commonly used non‐invasive modalities for imaging PVAT.

**TABLE 2:** Imaging modalities for perivascular adipose tissue visualisation

Imaging modality	Availability	Cost	Spatial resolution	Quantitative measures	Qualitative measures	Comments
CT	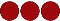	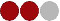	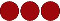	Volumetric assessment of PVAT around small, medium and large vessels	• Attenuation • FAI_PVAT_	• Radiation and contrast agent exposure
MRI	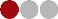	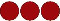	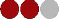	Volumetric assessment of PVAT around large vessels	• Proton density fat fraction	• Long scanning duration • No radiation
PET	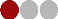	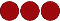	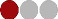	—	• SUV uptake	• Functional assessment of inflammatory/metabolic activity • Radiation exposure

*Note*: One red circle indicates ‘low’, two red circles ‘medium’ and three red circles ‘high’.

Abbreviations: FAI_PVAT_, perivascular adipose tissue fat attenuation index; PVAT, perivascular adipose tissue; SUV, standardised uptake value.

**FIGURE 7:**
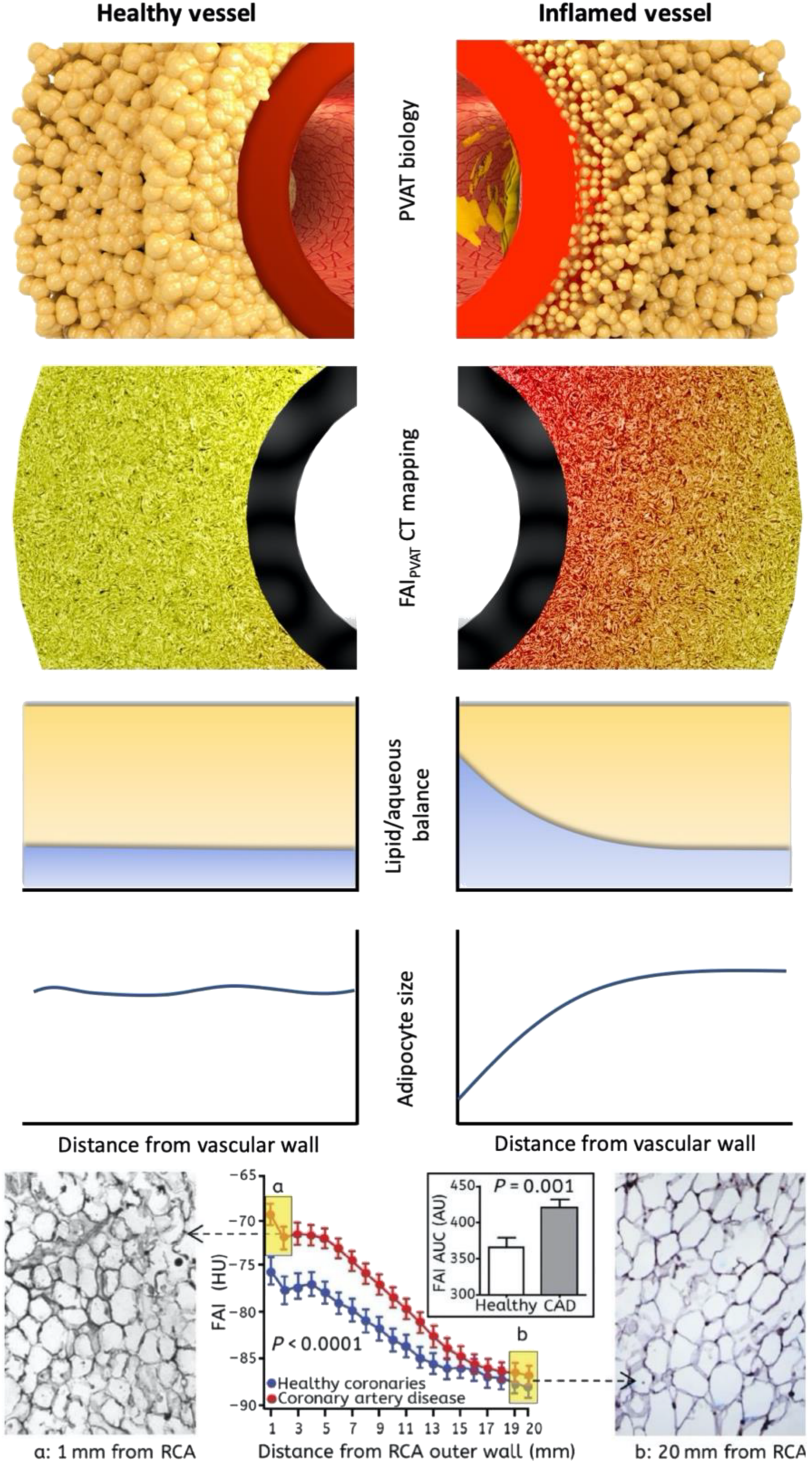
Illustrative visualisation of the perivascular fat attenuation index (FAI) gradient and radial distance from vascular wall in patients with coronary artery disease (CAD). PVAT, perivascular adipose tissue; RCA, right coronary artery. Parts reproduced with permission from Antonopoulos et al. (2017)

Attenuation values in PVAT around the coronaries are affected by inflammatory signals coming from the vascular wall that induce structural changes at a microscopic level, resulting in a gradient of adipocyte size in the first few millimetres around the inflamed coronary arterial wall. This gradient causes attenuation shifts between a more aqueous/less lipophilic phase close to the inflamed artery and a less aqueous/more lipophilic phase in the non‐PVAT within the epicardial fat (Antoniades, Antonopoulos, & Deanfield, 2019) (Figure 7). In healthy PVAT, attenuation values directly next to the vascular wall are driven towards the more negative values within the widely accepted Hounsfield unit (HU) range (−190 to −30 HU) for adipose tissue characterisation in CT, thanks to large, fully differentiated adipocytes with high lipid content. However, the presence of vascular inflammation is accompanied by small, less differentiated adipocytes, with low lipid content directly adjacent to the vascular wall, that force attenuation to rise to the less negative values (towards the −30‐HU end). This reflects the shift in tissue composition that is governed by the following elements: ‐ (i) reduced intracellular lipid content, (ii) increased intracellular aqueous phase, which replaces intracellular lipids after lipolysis, (iii) increased extracellular fluid from the shrinking adipocytes and (iv), oedema in the inflamed environment. This important observation led to the creation of the perivascular Fat Attenuation Index (FAI), an imaging tool that measures weighted three‐dimensional attenuation gradients of adipose tissue in the perivascular space (Antonopoulos et al., 2017).

## 5 FAI_PVAT_ CALCULATION

The calculation of perivascular Fat Attenuation Index (FAI_PVAT_) involves the use of artificial intelligence‐enhanced algorithms (CaRi‐HEART, Caristo Diagnostics, Oxford, UK) that provide accurate and reproducible weighted measures of attenuation in concentric 1‐mm three‐dimensional layers of perivascular tissue around the human arterial wall (Antoniades, Antonopoulos, & Deanfield, 2019). The calculation process is complex, as it includes multiple analysis steps. First, the heart is segmented, the vessel of interest is delineated in a fully automated way by the CaRi‐HEART application and subsequently, the perivascular space is defined and the adipose tissue is analysed in multiple layers in the perpendicular dimension. These analyses are performed within the standard adipose tissue HU range window of −190 to −30 HU by algorithms that involve multiple adjustments that differentiate FAI_PVAT_ from crude ‘mean CT attenuation (or radiodensity)’, because it is appropriately corrected and weighted for factors related with technical CT scan characteristics, the local coronary anatomy, the background adipocyte size and others, interpreted using machine learning‐enhanced modelling. Correcting attenuation values is of high importance in interpreting coronary inflammation. Obesity is known for hyperplasia and hypertrophy of adipose tissue, and obese patients have overall larger adipocytes that could falsely reduce the mean and underestimate vascular inflammation. Mean values are also subject to technical aspects of the scan, such as tube voltage and reconstruction algorithms, all of which are corrected in the CaRi‐HEART algorithm (Antoniades, Kotanidis, & Berman, 2019). Lastly, FAI is independent of signals intrinsic to the vessel, such as arterial calcification or lumen attenuation (Antonopoulos et al., 2017).

## 6 FAI_PVAT_ AS A MARKER OF BACKGROUND GLOBAL INFLAMMATION

FAI_PVAT_ can be used to track background inflammation present in the coronary tree of patients at high risk of developing atherosclerotic plaques. The initial studies of FAI_PVAT_ were confined to the proximal 40‐mm sections of the three major coronary arteries: ‐ right coronary artery (RCA), left anterior descending artery (LAD) and left circumflex artery (LCX) (Antonopoulos et al., 2017), due to lack of molecular validation of FAI measurements in other coronary segments. We found that using this standardised measurement of perivascular FAI in the proximal 40‐mm segments of the big coronaries yields provides an excellent surrogate of the background vascular inflammatory burden of the entire coronary tree—independently of the presence of plaque—which may be abnormal even in the absence of any visible coronary atherosclerotic plaque (Antoniades, Antonopoulos, & Deanfield, 2019). Perivascular FAI measured in these standardised proximal coronary segments is significantly higher in patients with coronary artery diseas compared with individuals with nonatherosclerotic vessels (Movie S1). It also remains independent of local coronary calcification or overall coronary calcium score (CCS), after adjusting for age, gender and other cardiovascular risk factors (Antonopoulos et al., 2017).

## 7 FAI_PVAT_ AS A MARKER OF LOCAL CORONARY PLAQUE INFLAMMATION AND VULNERABILITY

**FIGURE 8:**
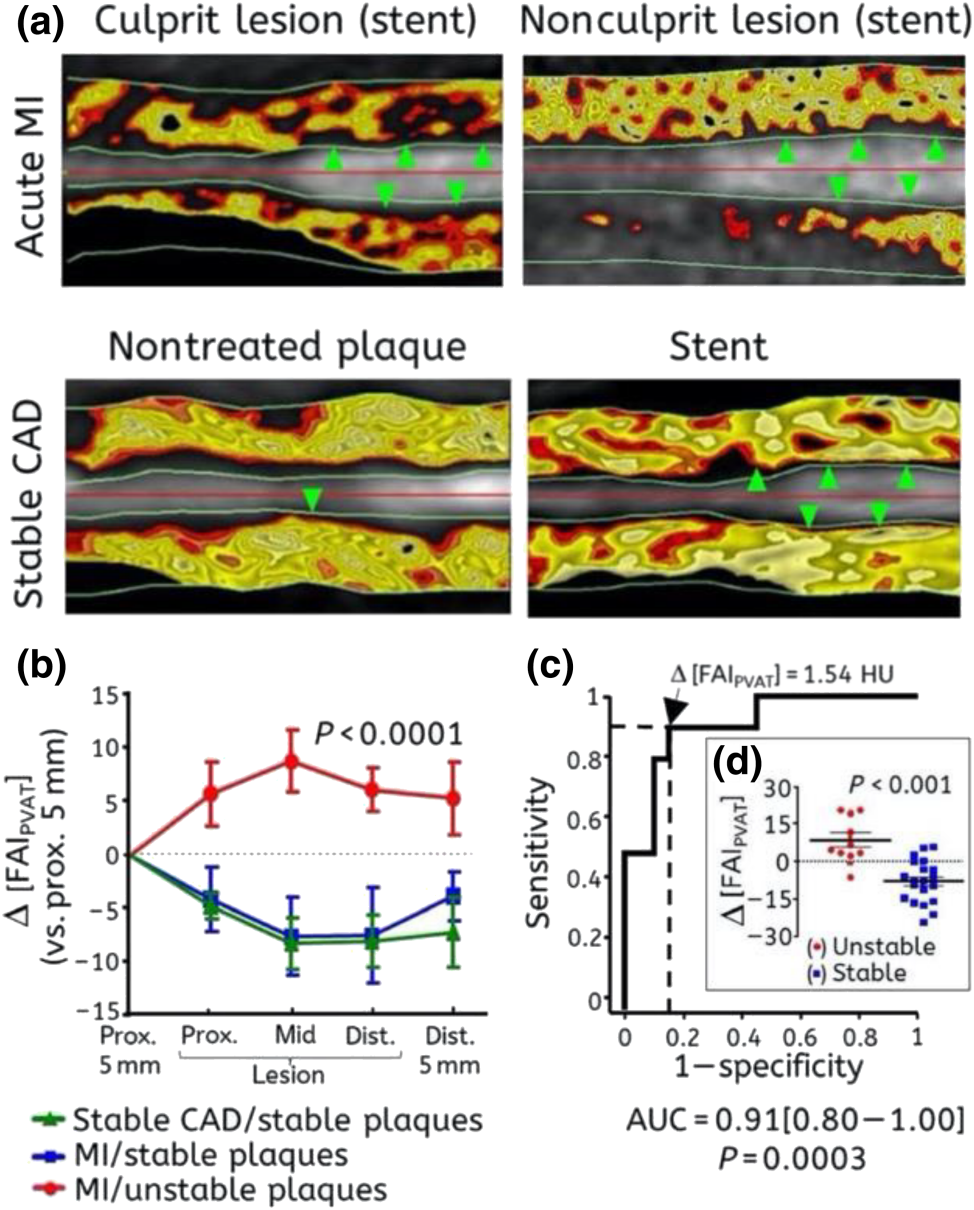
(a) Representative images of delineated perivascular fat with fat attenuation index (FAI) coloured mapping around (i) a culprit lesion (green arrows) demonstrating abnormal FAI in a patient with acute myocardial infarction (MI) (upper left image), (ii) a nonculprit lesion (green arrows) from the same patient also showing abnormal FAI (upper right image), (iii) a stable atherosclerotic lesion without a stent (single green arrow, lower left image) and (iv) a stent (green arrows) implanted at least 3 months before imaging (lower right image); black colour indicates nonadipose tissue with attenuation values outside the −30 to −190‐HU range. (b) Perivascular FAI changes around ruptured (culprit) atherosclerotic lesions (*n* = 10) of patients with acute MI, nonculprit lesions of the same patients (*n* = 7) or lesions of stable coronary artery disease (CAD) patients (*n* = 13). (c, d) Δ[FAI] comparison between stable and unstable plaques and receiver operating characteristic (ROC) curve analysis for its diagnostic accuracy in the detection of unstable plaques (culprit lesions). HU, Hounsfield units; Δ[FAI], FAI (around lesion) − FAI (proximal segment). Reproduced with permission from Antonopoulos et al. (2017)

Apart from this standardised analysis, PVAT phenotyping with FAI_PVAT_ may be performed around atherosclerotic plaques themselves and has the potential to yield informative results on local variability of coronary inflammation and plaque vulnerability. Corrections for background inflammation present in the coronary tree are done by referencing the change of perivascular FAI around the vulnerable ‘inflamed’ plaque to a segment proximal to the lesion. FAI is significantly increased around the culprit lesions in patients with acute coronary syndrome as compared with non‐culprit lesions of the same patient or around stable lesions in stable patients (Antonopoulos et al., 2017) (Figure 8). Interestingly, perivascular FAI around the culprit lesion returns back to baseline values in the months following an acute event, proving its dynamic ability to detect localised shifts in coronary inflammation as it reaches higher values in the period leading up to rupture (Antonopoulos et al., 2017). Other studies have validated these findings with Goeller et al. (2018) reporting an increase in crude attenuation values around vulnerable plaques, whereas increased plaque inflammation assessed by an increase in ^18^F‐NaF PET uptake on PET‐CT imaging has been shown to strongly correlate with higher perivascular fat radiodensity in stable patients with high‐risk plaques (HRPs) on CTA (Kwiecinski et al., 2019). Finally, high PVAT radiodensity close to the vascular wall has been linked with another coronary inflammatory condition, spontaneous coronary dissection (Hedgire et al., 2018), validating the proof of principle that a link exists between coronary artery inflammation and PVAT phenotype.

## 8 FAI_PVAT_ TO MONITOR RESPONSIVENESS TO TREATMENTS

**FIGURE 9:**
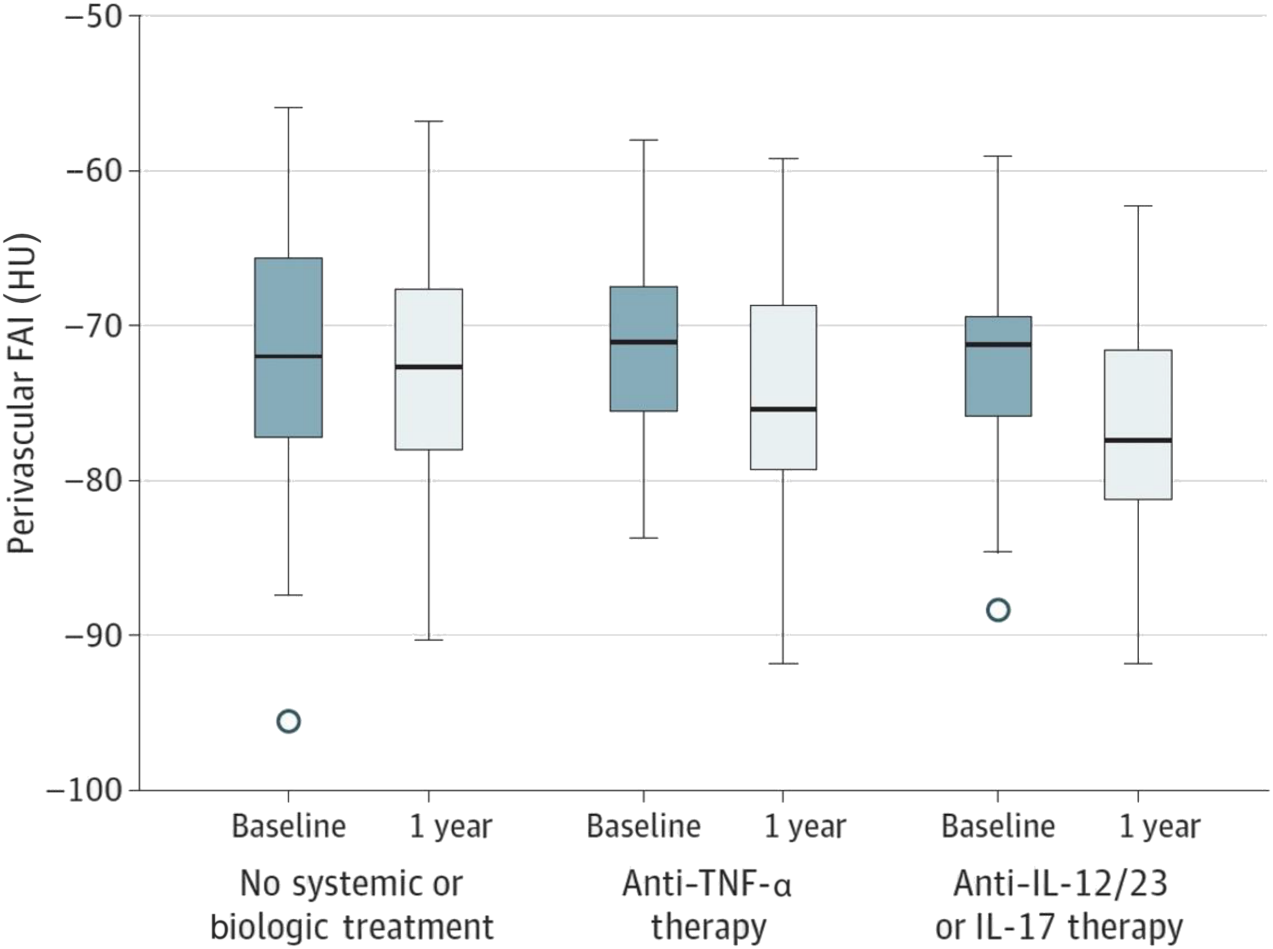
Changes in perivascular fat attenuation index (FAI) in patients with psoriasis not receiving any systemic or biological therapy during 1 year or receiving biological therapy (*P* = 0.99) during 1 year with anti‐TNF‐α therapy (*P* < 0.001) or anti‐IL‐12/23 and anti‐IL‐17 therapies (*P* < 0.001). HU, Hounsfield units. Reproduced with permission from Elnabawi, Oikonomou, et al. (2019). American Medical Association. All rights reserved. Colour images are available online

Being an excellent surrogate of coronary inflammation, FAI_PVAT_ can be utilised as a non‐invasive, approachable and achievable marker to directly interrogate changes in coronary inflammation, following anti‐atherogenic and anti‐inflammatory treatment. Perivascular FAI measured both around culprit lesions and the right coronary artery (RCA) in coronary CT angiography (CCTA) scanning performed within 96  hours of admission for acute myocardial infarction changed dynamically at 6  months, with significant changes being detectable as early as 5 weeks after the event following initiation of optimal secondary preventative therapies (Antonopoulos et al., 2017; Oikonomou et al., 2019). Furthermore, in an observational cohort of 134 patients with moderate to severe psoriasis but low cardiovascular disease risk, treatment with anti‐inflammatory biological agents, including anti‐TNF‐α, anti‐IL17 and anti‐IL12/23 monoclonal antibodies, was shown to lead to a significant reduction in FAI_PVAT_ values at 1 year (Figure 9) compared with individuals treated with topical or UVB phototherapy (Elnabawi, Oikonomou, et al., 2019). Biological treatment, which had been previously shown to lead to reduction in noncalcified plaque burden and necrotic core in psoriasis (Elnabawi, Dey, et al., 2019), can also be tracked for its vascular inflammation‐lowering abilities with FAI_PVAT_ (Movie S1). Finally, a more recent study including 108 patients referred for statin treatment observed a significant reduction in FAI_PVAT_ around noncalcified as well as mixed plaques (−68.0 ± 8.5 vs. −71.5 ± 8.1 HU, *P* < 0.001 and −70.5 ± 8.9 vs. −72.8 ± 9.0 HU, *P* = 0.014, respectively) in follow‐up CCTA at 1 year (Dai et al., 2020).

## 9 FAI_PVAT_ AND LONG‐TERM OUTCOMES

**FIGURE 10:**
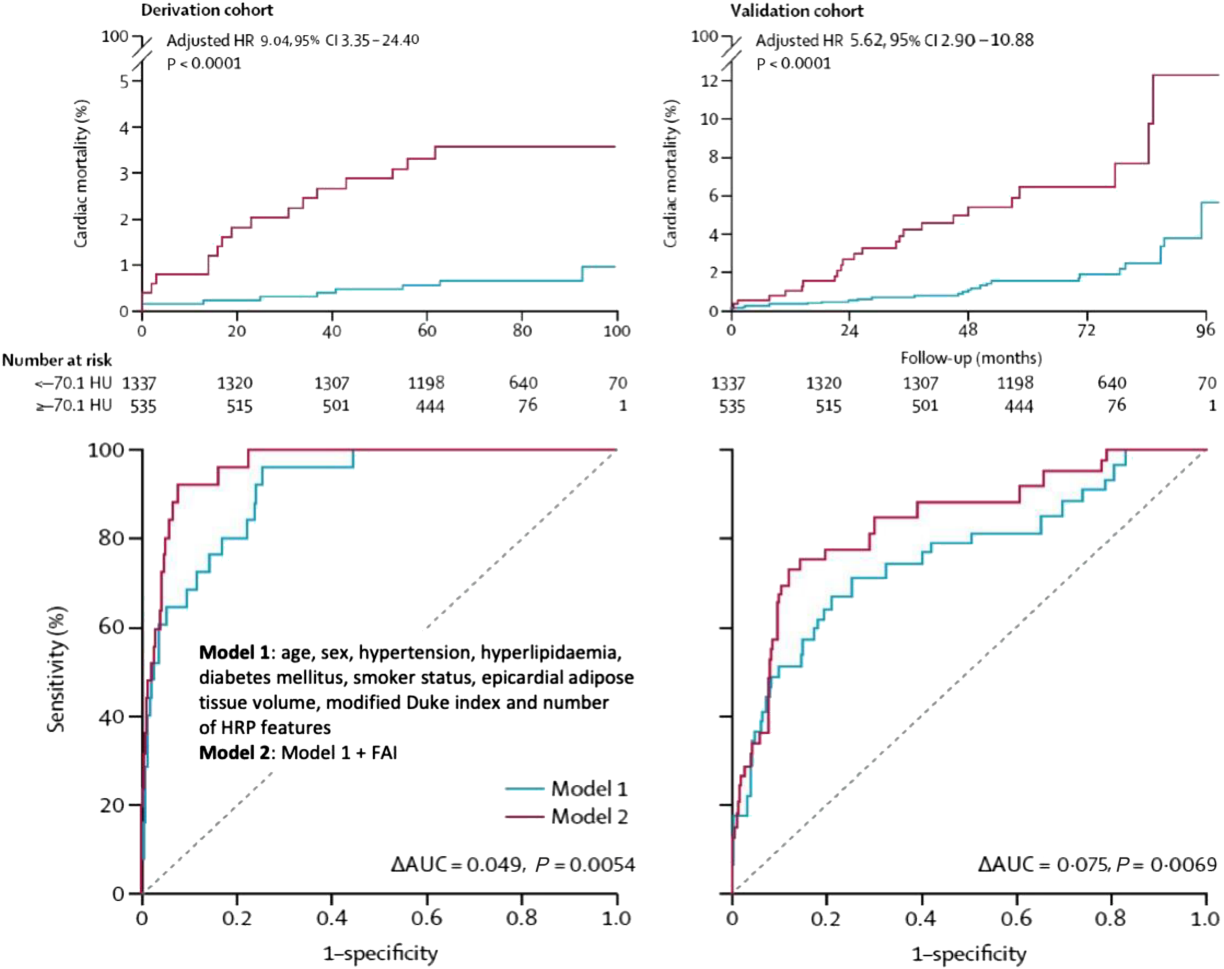
Kaplan–Meier curves of cardiac mortality with high versus low perivascular fat attenuation index (FAI) for the derivation and validation cohorts. Hazard ratios (HRs) are adjusted for risk factors, technical factors, the extent of coronary artery disease and number of high‐risk plaque features. Comparison of time‐dependent ROC curves (at 6 years) and respective area under the curve (AUC) of two nested models for discrimination of cardiac mortality in the derivation and validation cohorts. Model 1 represents the current state of the art in risk assessment and consists of age, sex, risk factors (hypertension, hypercholesterolaemia, diabetes mellitus, smoker status and epicardial adipose tissue volume), modified Duke coronary artery disease index and number of high‐risk plaque features on coronary CTA. Model 2 incorporates perivascular FAI values into Model 1. HRP, high‐risk plaque; HU, Hounsfield units. Reproduced with permission from Oikonomou et al. (2018)

In an effort to explore the predictive value of FAI_PVAT_ to predict long‐term clinical outcomes, the CRISP‐CT (Cardiovascular RISk Prediction using Computed Tomography) study was designed with almost 4000 individuals undergoing coronary CT angiogram (CCTA) as part of their clinical care in Erlangen (Germany) and Cleveland (USA) (as derivation and validation cohorts, respectively, with ~2000 individuals each) (Oikonomou et al., 2018). Perivascular FAI around the proximal segment of the LAD and RCA was strongly predictive of all‐cause and cardiac mortality but not noncardiac mortality (Oikonomou et al., 2018). Individuals with high perivascular FAI had a hazard ratio (HR) (95% confidence interval [CI]) 2.55(1.65–3.92) in the derivation and 3.69 (2.26–6.02) in the validation cohort for all‐cause mortality. In terms of cardiac mortality alone, individuals with high FAI_PVAT_ had ninefold higher risk in the derivation cohort and 5.6‐fold higher risk in the validation cohort. Of note, addition of FAI_PVAT_ to a model that includes clinical risk factors, calcium score, the extent of coronary atherosclerosis and the presence of high‐risk plaques features improved performance, proving that FAI_PVAT_ offers incremental predictive values on top of the current state of the art in risk assessment using CCTA (ΔAUC = 0.049, *P* = 0.0054 in the derivation cohort and ΔAUC = 0.075, *P* = 0.0069 in the validation cohort) (Figure 10). This permits reclassification of an individual's risk, independently of their background calcium score, the presence of coronary artery disease or any features (Desai, 2018).

**FIGURE 11:**
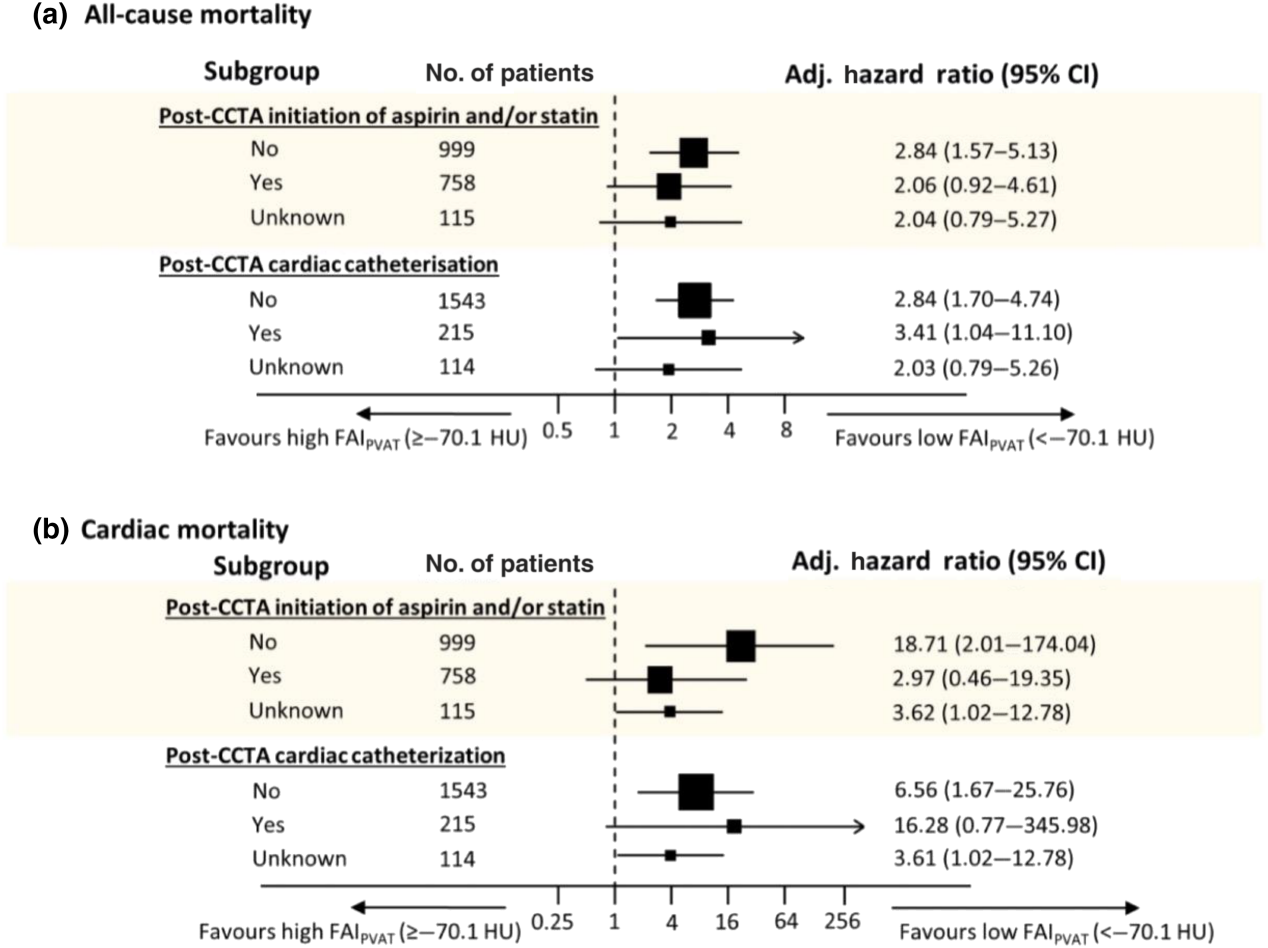
Subgroup analysis based on clinical recommendation for treatment with statin and/or aspirin after coronary CT angiography (CCTA) about the predictive value of perivascular fat attenuation index (FAI) for all‐cause (a) and cardiac mortality (b). Cox regression models were adjusted for age, sex and epicardial adipose tissue volume. FAI was strongly associated with cardiac mortality events in patients that did not receive recommendations for treatment with statins or aspirin, whereas the association in the group that did receive such recommendations was non‐significant. This highlights a potential role for FAI in secondary prevention measures and personalised selection of patients that can qualify for medical treatment after screening in current clinical practice. CI, confidence interval. Reproduced with permission from Oikonomou et al. (2018)

Another significant finding stemming from the CRISP‐CT study was that individuals with high FAI_PVAT_ but absence of high‐risk plaque features exerted a higher cardiac risk as opposed to patients with high‐risk plaque features but low FAI_PVAT_ values, highlighting yet again that FAI_PVAT_ is a robust, independent biomarker that describes aspects of vascular biology not detectable by other indices (Oikonomou, Desai, et al., 2020). Subgroup analyses performed in the CRISP‐CT study revealed that perivascular FAI measured at baseline was no longer predictive of cardiac mortality (adjusted HR 2.97, 95% CI 0.46–19.35; *P* = 0.25) in individuals who received a clinical recommendation to initiate statins or aspirin following clinical CCTA. In contrast, amongst those who did not initiate any such treatment after their scan, perivascular FAI maintained its predictive value for cardiac mortality (adjusted HR 18.71, 95% CI 2.01–174.04; *P* = 0.01) (Figure 11). This observation suggests that the risk identified by perivascular FAI is modifiable and could potentially be tracked by repeat CCTA after treatments initiation (Tuñón et al., 2019). This finding was later validated in an independent cohort patients treated with inflammation modifying agents for psoriasis, in which biological therapy with anti‐TNF‐α, anti‐IL12/23 or anti‐IL17 antibodies was associated with a reduction in FAI values used to assess coronary inflammation as discussed above (Elnabawi, Oikonomou, et al., 2019).

**FIGURE 12:**
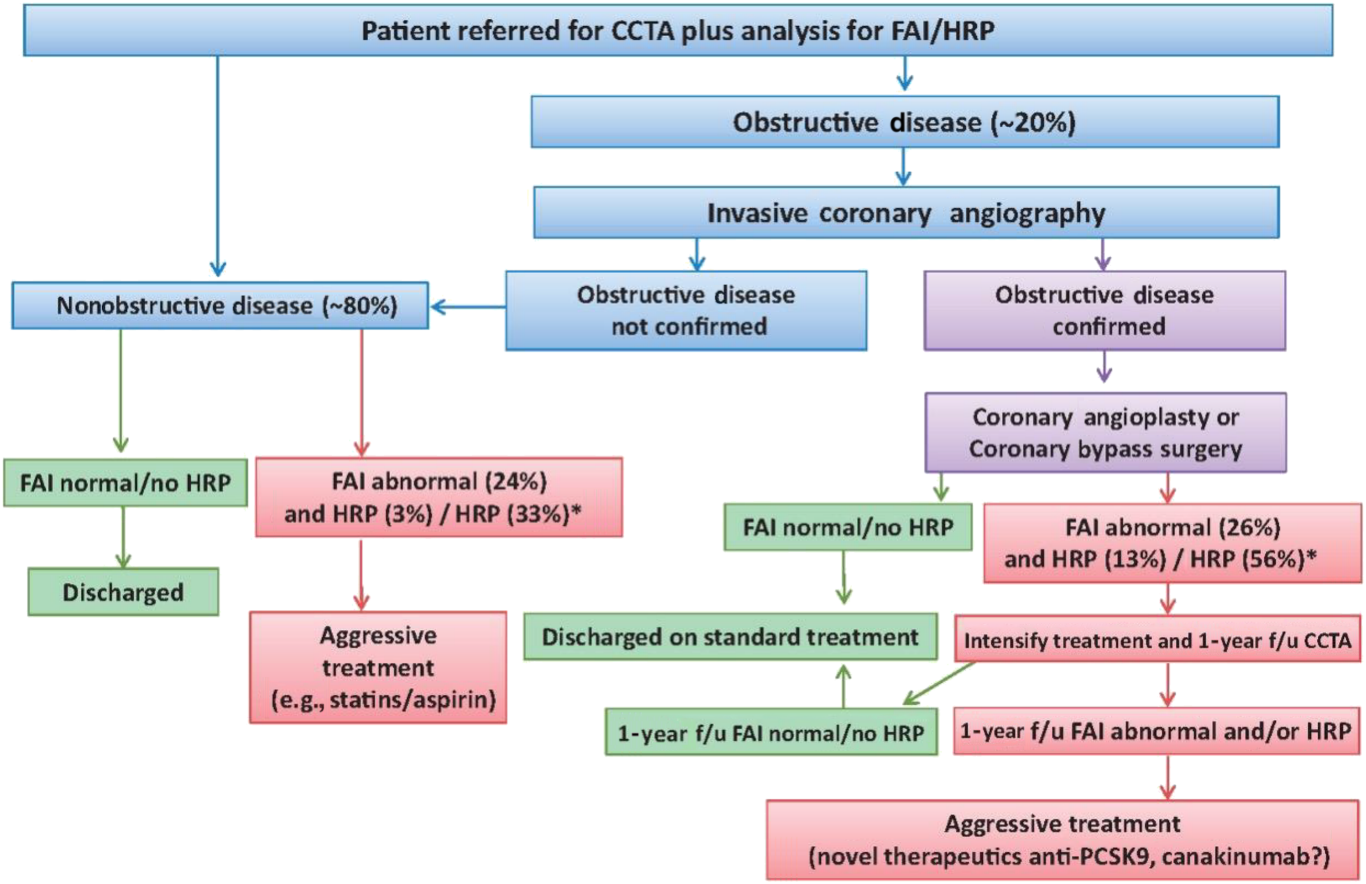
Vision for implementing fat attenuation index (FAI) and high‐risk plaque (HRP) features in clinical practice. anti‐PCSK9, pro‐protein convertase subtilisin/kexin type 9 inhibitors; CCTA, coronary CT angiography; f/u, follow‐up. ^*^% referred to data from CRISP‐CT study. Reproduced with permission from Antoniades, Kotanidis, and Berman (2019)

It becomes evident that FAI_PVAT_ can be utilised not only for disease classification but also for management decision making. We have proposed a decision model that highlights our vision as to how the assessment of coronary inflammation with FAI_PVAT_ can be incorporated in clinical practice (Figure 12). This approach is highly personalised and could facilitate the selection of patients for the use of high‐cost therapies, such as monoclonal antibodies or PCSK9 inhibitors.

## 10 PVAT RADIOMIC PHENOTYPING

**FIGURE 13:**
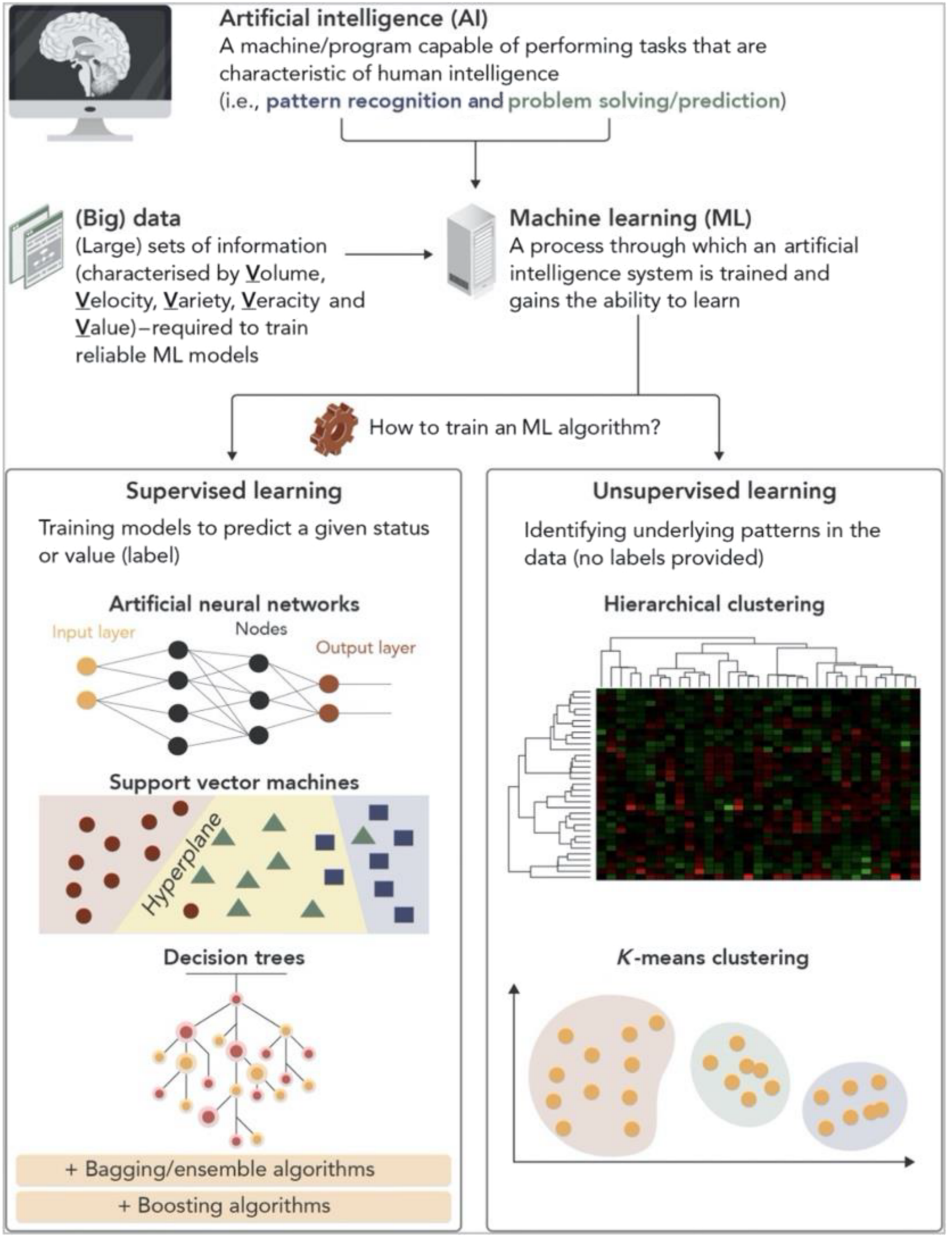
Schematic presenting the different categories of radiomic features extracted from a segmented volume of interest and basic big data terminology. Higher order features reflect the unique spatial arrangement of voxels, rather than simple voxel attenuation distribution derived from histogram‐based first‐order features. Artificial intelligence (AI) describes a program that mimics ‘cognitive’ functions that are associated with natural intelligence displayed by humans and animals. Machine learning refers to the process through which an AI system is trained to perform ‘cognitive’ functions. There are two broad types of learning used in medicine: supervised, in which different algorithms are used to predict the class or value of a known label, and unsupervised, in which the algorithm itself selects and attributes classes based on the dataset patterns. Reproduced with permission from Oikonomou, Siddique, et al. (2020)

As we slowly move towards a big data‐dominated era across all facets of scientific research, advances in computational and imaging processing methodologies have rendered medical imaging an ideal field that offers a promising pool of large datasets and registries. Imaging studies in oncology have introduced radiomics to the medical research world with their presence in publication records within cardiovascular research steadily increasing. Radiomics refers to the big data field that focuses on extraction of large amounts of quantitative data from images and their use for machine learning modelling. Specialised data characterisation algorithms are able to identify patterns of data behaviour within the dataset of numbers that comprise an image or a segmented volume, which cannot possibly be perceived by the human naked eye (Gillies et al., 2015). These patterns are not abstract but rather may be linked with underlying pathology. Imaging studies in cancer patients have linked specific shape‐ and texture‐related patterns within the volume of the tumour to its phenotype and also overall clinical prognosis (Aerts et al., 2014). Figure 13 presents a schematic of basic radiomic features and analytical techniques employed in radiomics (Oikonomou, Siddique, & Antoniades, 2020).

**FIGURE 14:**
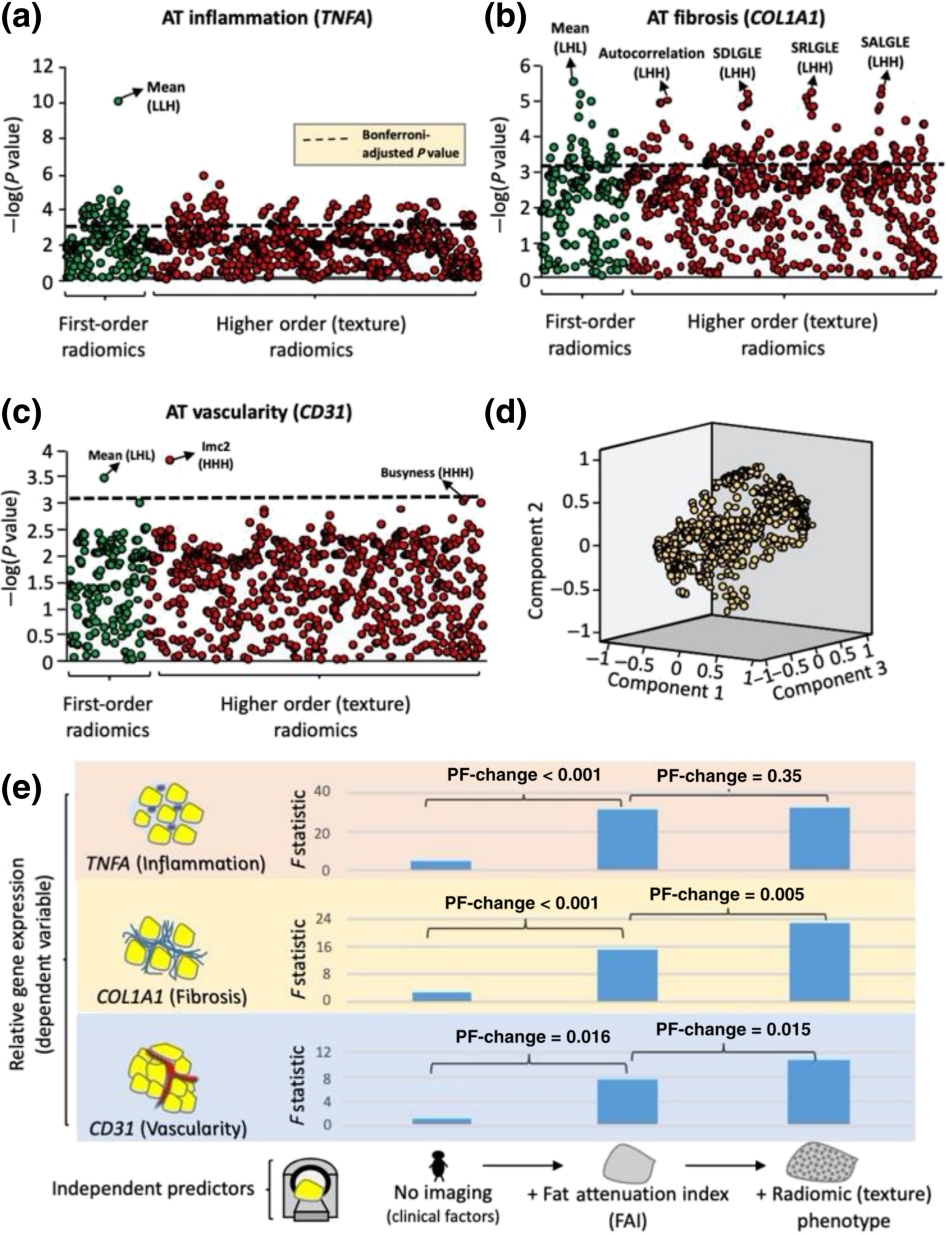
(a–c) Manhattan plots presenting the strength of association between adipose tissue (AT) radiomic features and the relative gene expression of *TNFA* (inflammation), *COL1A1* (fibrosis) and *CD31* (vascularity). (d) Component plot of the three principal components of the AT radiome. (e) Comparison of nested linear regression models with relative gene expression as the dependent variable and (i) clinical risk factors alone (Model 1: age, sex, hypertension, hypercholesterolaemia, diabetes mellitus and body mass index); (ii) Model 1 + mean attenuation (Model 2); and (iii) Model 2 + PVAT radiome (first three principal components) as the independent predictors. Imc, informational measure of correlation 2; L/H, low/high wavelet transformation; SALGLE, small‐area low grey‐level emphasis; SDLGLE, small dependence low grey‐level emphasis; SRLGLE, short‐run low grey‐level emphasis. Reproduced with permission from Oikonomou et al. (2019)

Cardiac imaging research has also started utilising tools offered by radiomics (Dey & Commandeur, 2017). A recent study focusing on vascular wall and plaque characterisation has shown that the metabolic activity of lesions with the napkin‐ring high‐risk plaques can be stratified by radiomic features within the arterial (Kolossváry et al., 2017). We recently performed a large radiotrancriptomic experiment with human adipose tissue biopsies so as to validate the link between radiomics and biology. We tested a range of radiomic features and how they correlate with three major aspects of adipose tissue biology: inflammation—using the expression of TNF‐α gene as marker; fibrosis—evidenced by collagen (COL1A1 gene) expression; and vascularity—reflected by the expression of endothelial marker CD31 (*PECAM1* gene). As expected, TNF‐α expression was most significantly linked with features related mostly to tissue attenuation (captured by the FAI), confirming the concept that attenuation first‐order metrics are descriptive of inflammation. Fibrosis and microvascular remodelling were also found to correlate with differential groups of radiomic features mostly inherent to tissue homogeneity and texture, suggesting that radiomic phenotyping can provide a comprehensive evaluation of the biological variation within adipose tissue (Oikonomou et al., 2019) (Figure 14).

**FIGURE 15:**
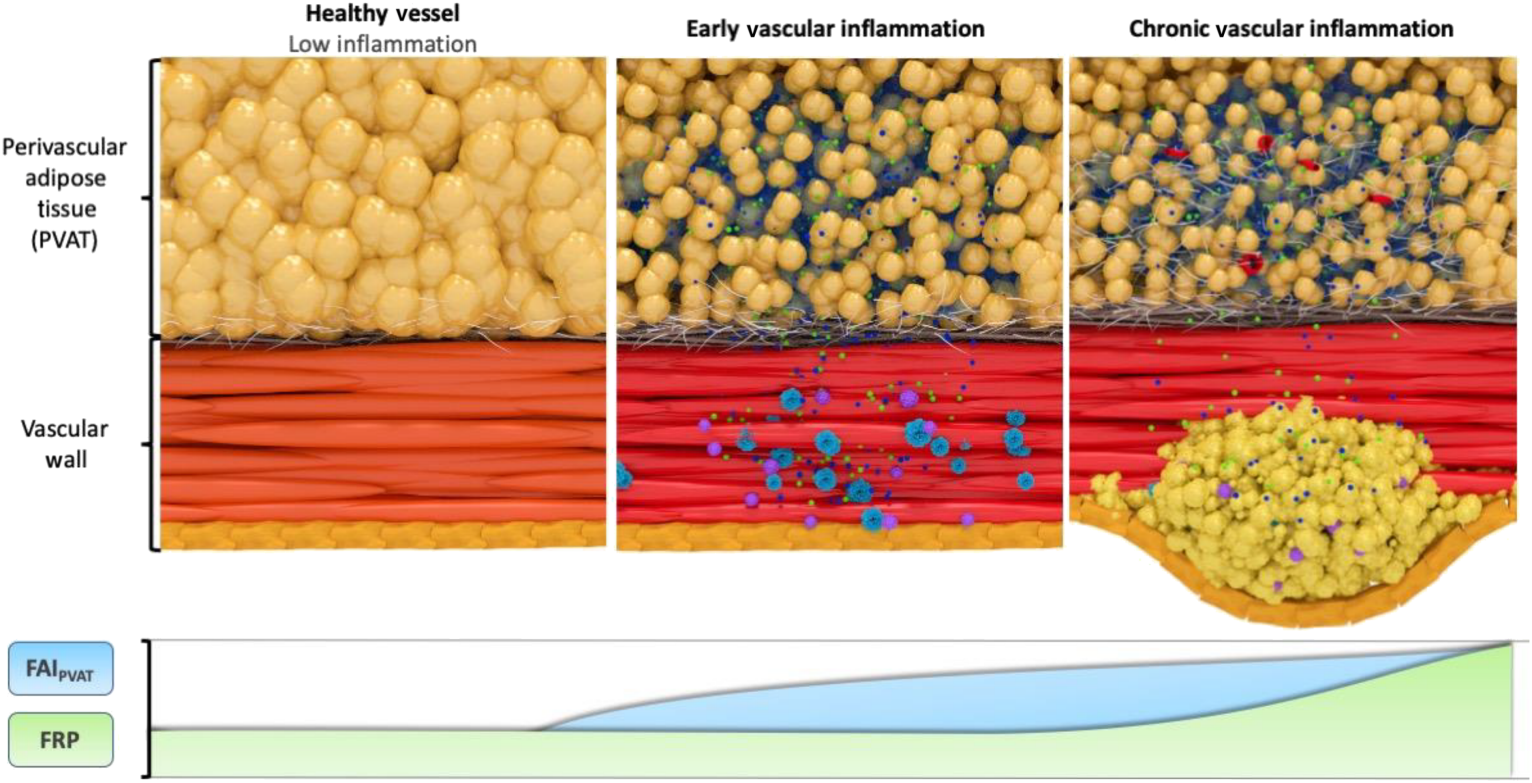
Schematic representation of the biology underlying fat attenuation index (FAI) and fat radiomic profile (FRP). Coronary inflammation drives phenotypic changes in perivascular adipose tissue (PVAT), including decreased adipocyte size, differentiation and intracellular lipid content. The novel CT‐derived FAI captures weighted three‐dimensional attenuation values reflecting biological phenotypic gradients inherent to coronary inflammation. Persistence of vascular inflammation and atherosclerotic disease may lead to further changes in PVAT composition, characterised by increased extracellular fibrosis and neomicrovascularisation. Those changes can be detected by radiomic phenotyping of PVAT using the novel signature FRP

With these initial observations in mind, we developed a CT‐specific radiomic signature within the imaging profile of coronary PVAT that incorporates elements of not only inflammation but also permanent fibrotic and microvascular changes that would enable increased cardiac risk detection. We then applied this new signature, referred to as the fat radiomic profile (FRP), to a population of 1575 individuals from the CCTA arm of the SCOT‐HEART trial. Interestingly, patients with high FRP had a striking 11‐fold higher risk for major adverse cardiovascular events, independently of traditional risk factors, presence of coronary artery disease (≥50% stenosis), presence of high‐risk plaque features, Agatston coronary calcium scoring and scanner type (Oikonomou et al., 2019). Importantly, FRP was able to capture residual cardiovascular risk in this population offering incremental prognostic information beyond current CCTA‐based tools (including Agatston CCS, high‐risk plaque features and luminal stenosis). Testing FRP on serial CCTA scans from patients with an acute event, it was shown that 6 months after the index event FRP remained unchanged with FAI significantly decreasing towards baseline values, as discussed above, suggesting that although FAI is a dynamic inflammatory burden marker, FRP can detect more permanent structural changes in PVAT (Oikonomou et al., 2019) (Figure 15). However, as with FAI, the reversible or modifiable nature of FRP has yet to be determined in clinical trials or observational studies. Treatment with biologics or even standard of care may have the potential to reverse aforementioned changes in PVAT.

## 11 CONCLUSIONS

Robust advances in technology and computing have brought the field of medical imaging to the fore, enabling comprehensive tissue characterisation on the molecular level. CT lies at the centre of imaging in cardiology, having recorded rapid advances during the past few years in plaque and adipose tissue phenotyping. PVAT is a unique structure, with a highly diverse secretome, that is implicated in a bidirectional interplay with the adjacent vascular wall, both affecting and being affected by aspects of its biology, mainly vascular inflammation. The latter induces molecular, transcriptional and structural alterations to PVAT, changing its macroscopic phenotype, which is characterised by concentric three‐dimensional gradients of small adipocytes with low lipid content, interstitial space expansion, oedema, fibrosis and neovascularisation, around inflamed vessels. Non‐invasive CT imaging can track these phenotypic changes, by the detection of three‐dimensional attenuation and texture shifts in the visualised adipose tissue. This fundamental observation has led to the creation of the perivascular FAI (FAI_PVAT_), an artificial intelligence‐enhanced biomarker, that captures weighted attenuation gradients in the perivascular space inherent to coronary inflammation, providing an excellent surrogate of both baseline inflammatory burden in the vasculature as well as localised inflammation around the vulnerable plaque. The ability of FAI_PVAT_ to track vascular inflammation may have significant implications in assessing vascular involvement outside of the usual disease spectrum a cardiologist comes across, such as in the case of the most recent COVID‐19 pandemic (Guzik et al., 2020). The science behind FAI_PVAT_ has been patented by the University of Oxford and its technology is being commercialised by Caristo Diagnostics, a spin‐out company of the University of Oxford that aims to deliver imaging software to assist clinicians in making personalised treatments decisions related to a patient's future cardiac management.

Further, PVAT radiomic profiling has also emerged lately as a highly sophisticated analytical method of texture analysis, describing different aspects of the inflammatory structural changes in PVAT, such as fibrosis and neovascularisation. The development of custom‐made radio‐transcriptomic signatures tailored around specific inflammatory or other molecular pathways in the vascular wall who can affect PVAT's texture may transform the use of cardiac imaging and particularly cardiac CT, from a purely diagnostic to a prognostic clinical tool, with major clinical implications.

### 11.1 Nomenclature of targets and ligands

Key protein targets and ligands in this article are hyperlinked to corresponding entries in the IUPHAR/BPS Guide to PHARMACOLOGY http://www.guidetopharmacology.org and are permanently archived in the Concise Guide to PHARMACOLOGY 2019/20 (Alexander et al., 2019).

## CONFLICT OF INTEREST

The methods for analysis of the perivascular fat attenuation index described in this review are subject to Patent PCT/GB2015/052359 and Patent Applications PCT/GB2017/053262, GB2018/1818049.7, GR20180100490 and GR20180100510, licenced through exclusive licence to Caristo Diagnostics. C.A. is a founder and shareholder of Caristo Diagnostics Ltd., a CT image analysis company.

## Supporting information


**Movie S1.** Supporting information
